# English Tube Teeth: Their Use in Plate-, Crown-, and Bridge-Work

**Published:** 1893-10

**Authors:** John Girdwood

**Affiliations:** L.D.S. (Edin.), D.D.S. (Univ. of Penna.), Edinburgh, Scotland


### ENGLISH TUBE TEETH: THEIR USE IN PLATE-, CROWN-, AND BRIDGE-WORK.[1]

BY JOHN GIRDWOOD, L.D.S. (EDIN.), D.D.S. (UNIV. OF PENNA.), EDINBURGH, SCOTLAND.

THE absence of a detailed description of the method of using tube teeth from any of the standard American works on operative or mechanical dentistry has long been a subject of surprise to me. It still puzzles many of the leading dentists in Europe, and particularly in England, where tube teeth are still largely used, and found,

---

[1] Read before the World's Columbian Dental Congress, Chicago, August, 1893.

where judiciously employed, superior to flat teeth, to know why the system universally practised here before the introduction of vulcanite should have failed to attract any notice in America.

The present seems a fitting time to bring before you this subject, especially when we know that in crown and bridge-work, specialties indigenous to America, these teeth have a new field opened to them.

The English tube tooth differs from the flat favorite in one essential,—viz., that its attachment to the piece to which it is adjusted is effected by means of a central tube (running through the body of the tooth), and into it a pin or post is introduced.

Before proceeding further, it may here be well to set before you a few of the advantages claimed for tube over flat teeth.

*First.* Although adaptable in any situation on both jaws, they are specially superior as masticators. They are much stronger than their flat rivals. The tube tooth is supported over its whole lower surface, and the greatest strain in occlusion falls mostly in a vertical direction upon the crown, whereas in a flat tooth the impact of the bite is more evenly distributed.

*Second.* They allow of easy removal for repair.

*Third.* In soldering the backings of flat teeth to the plate, the danger of warpage is great. By the use of tube teeth this risk is entirely removed.

*Fourth.* They are more adaptable; a very long tooth can be cut down to any length, and, the body being of the same texture throughout, can be ground and polished perfectly.

*Fifth.* They can be used for plate-, crown-, and bridge-work, and in some cases in combination with vulcanite. From their ease of adaptability, a small stock of these teeth goes a long way. Hence they are cheaper.

*Sixth.* Being a more faithful reproduction of the teeth they replace, they feel more comfortable to the tongue, and are less bulky.

*Seventh.* They are more easily kept clean, because backings are done away with, and better supports substituted, which being surrounded by porcelain are out of the reach of any impurity.

For crown- and bridge-work they have all the advantages already enumerated, and, in addition, they can be more perfectly and directly fitted to the root than any other form of porcelain crown. They retain when mounted for wear in the mouth their translucency and natural appearance, qualities so often destroyed by the gold backing.

There seems to me, however, to be one defect in the tube incisors and cuspids as at present manufactured. In these the base is frequently too small antero-posteriorly, and, consequently, in many cases it is impossible with them to cover the root completely. Moreover, the tube is very often too near the front, thus destroying the axis of the crown with its root. The bicuspids and molars, however, are free from any such fault, and are pre-eminently adapted for crown-work.

The application of tube teeth requires the use of a special set of simple hand tools which had best be described here.

A counter-sink for clearing away the burr which forms upon the end of the tube when ground, and for slightly enlarging the orifice of the tube at its base.

A tube file used to remove the *débris* from the tube after grinding.

A marker. This is a piece of straight round wire which should fit the tubes easily, but not loosely, and have one end filed almost to a central point.

A pair of flat-pointed pliers with a longitudinal groove in them for holding the pin while it is inserted in its socket in the plate.

A sharp-pointed graver.

A length of gold pin-wire.

A pot of paint, made by mixing olive oil and vermilion.

Their respective uses will be best described in an explanation of the method of mounting the teeth for which they are required.

For this purpose we shall take, by way of illustration, a partial gold upper, where the lateral incisor and cuspid on the right side and all the grinding-teeth on both sides, except the second right upper molar, are absent. Having struck and fitted the plate in the ordinary way, and fitted the clasp, a tube tooth is now selected for each side. Care must be taken that the teeth chosen shall be longer than is apparently necessary, so that some tooth-substance may be to spare in fitting to the plate and bite. They are now roughly fitted in the positions they will occupy. The counter-sink removes the burr from the platinum tube at its ground end, and the tube file clears out all *débris* from end to end. Having seen that the tube is clean, replace the teeth on the plate and fasten them in their desired positions with hard wax. Now pass the marking wire *tipped with vermilion paint* down each tube till it touches the plate, where it will leave a mark showing the places at which the holes are to be drilled to receive the pins. Remove the teeth from the plate, taking care not to injure the color-mark. This undisturbed, take the sharp-pointed graver and make a slight pit for the drill; do not here take the plate from the model, but proceed to drill the holes for the pins, being heedful to keep the drill in every respect at the same angle as was made by the marker with the plate. By means of a broach the holes should be enlarged till they are just a little too small to receive the pin-wire full thickness; the rough edge or burr left by the drill must be removed by file and graver, and the hole slightly counter-sunk on both sides. A suitable length of gold pin-wire should now be cut, and the end which is to fit the socket in the plate should be slightly tapered, so as to fit tightly and project a little way through on the palatal surface. A slight groove may with advantage be made longitudinally on the tapered end of the wire, for it assists the solder to run more readily from the palatal to the lingual surface. The tapered end of the pin and the pin-hole are then touched with borax, and the wire fixed firmly in place by means of the pliers, attention being paid to its direction. The tooth is next tried on, and having ascertained that this particular point is correct, we proceed to solder the pins, drawing the solder through from side to side. It is not necessary to invest the plate for this purpose, for the tightness of the pin in its socket will support it sufficiently. In soldering, it is of the utmost importance that the smallest possible quantity of solder be used. When the plate has cooled, the flux is removed by boiling in pickle. From the palatal surface cut off the projecting end of the pin, and smooth it down with corundum wheel and graver till it is level with the plate. Now replace the plate on the model, and file down the pins till they accurately fit the bite. Place the teeth on the pins, and if the latter should have tilted in soldering, it will be at once seen, and may be corrected by grasping the pin close to the plate and bending as required.

Now comes the fine fitting of the teeth, done best with small wheels. Paint the plate where the tooth will touch it, and press the latter gently to place; it does not fit; remove it, and a small red mark will show where it is too long; grind off here, and having used counter-sink and tube file, try the tooth on its pin again, and continue the process of alternate trying on and grinding till a perfect fit is obtained. Now grind the coronal surfaces of the teeth to suit the occlusion of the bite, using the vermilion paint freely. Next set the cuspid and first bicuspid on the right side, and these finished, the first bicuspid on each side serving as a guide, the pins for the remaining teeth may be inserted at one soldering.

The teeth are then polished and the coronal ends of the pins are finished to show a rounded end, or ground to the bite according to the requirements of the case. This done, the plate is finished in the usual manner. Previous to fixing the teeth, a few shallow cuts are made in each pin with a fine file. When the teeth have been properly cleaned and freed from all traces of oil (which can be best done by boiling them for a few minutes in a strong solution of soda), their tubes are dried by cotton wound round a broach, and their interiors roughened by a clean tube file. The teeth are fixed with sulphur. This material is melted in a small porcelain Berlin cup till it is quite liquid, and is kept in this condition and held by an assistant. The operator himself grasps the plate firmly with the pliers in the left hand, and heats the whole carefully over the lamp. This must be done gradually, and the flame ought not to play on the porcelain. In the right hand he takes a wire spatula, and dipping it in the molten sulphur, conveys it to the heated plate and teeth repeatedly, till a surplus begins to show itself. The sulphur runs by capillary attraction under the teeth and along their pins, and when the whole has cooled it sets hard and the teeth are immovable. The excess of sulphur may be removed with a fine-pointed knife, and the polishing of the plate makes it ready for the mouth.

This description of the method of fitting tube teeth applies in every particular to every case, be it partial or full, upper or lower.

Besides the ordinary tube teeth, single gum teeth of this kind are to be had, and, when judiciously used, they prove as satisfactory as flat-backs.

*English Tube Crowns.*—In America, during recent years, attention has occasionally been called to English tube teeth in crown-work, although no details of their application have been given. This branch of practice, however, is common enough in England, although the usual method of fitting the tooth to a model, fixing pin and tooth together, and finally cementing them upon the root, is open to many objections.

An improvement made by my partner, Mr. John Stewart, L.D.S., of Edinburgh, is well worth a description. The method is as follows: The root is prepared in the usual manner. If part of it remain above the level of the gum, apply the rubber dam to one tooth on each side before excising, having first anæsthetized the gum by painting on a twenty-per-cent. solution of cocaine. If possible use a ligature in preference to a clamp for fixing the rubber, because the latter interferes with the bite when the pin comes to be adjusted. Push the rubber up as far as you can, for the reason that it is well to have the union of root and crown covered by the gum when the dam is taken off. Now drill the canal with a twist drill a shade larger than the diameter of the wire to be used as a post. If, as often happens in the first bicuspid, the canal be bifid, a piece of wire may be bent to fit into each canal, and to it the straight post should be soldered, or the straight pin may be "kneed" and an additional "leg" soldered to it. The post may be made of gold, platinum, or English dental alloy; the last I prefer. The post, where possible, should have a fine shallow thread cut on it, except where it emerges from the root to enter the crown. This part should not be impaired in strength even by a screw-thread.

Having selected a suitable tooth, fit it roughly to the root. Place the pin in the root and try on the crown; if it be much out of line with the other teeth, this fault must be put right, by bending the pin or by reaming the canal in the direction necessary, or by a combination of both operations. Now try on the tooth and the pin once more; if everything is right, groove the walls of the canal with a wheel-burr, mix the cement, and, placing a little in the canal and round the pin, force the latter to place with the pliers. While the cement is yet soft take the crown and slightly oil its base; slip it on to the pin, and before the cement sets insure its right position. It had best be held in place till the cement has set. Now, having taken off the crown, trim away the surplus cement from the face of the root. The face of the root may be cut out round the post, and filled with gold or amalgam, if thought desirable. Make the patient close his teeth, and grind the post till it is clear. Now fit the tooth on the root as you would to a plate, but instead of using vermilion paint for fine fitting use a small disk of thinnest articulating paper, and grind off where the tooth is marked by it till a perfect fit is obtained. Next grind to fit the bite margin, remove the excess of porcelain till the sides of root and crown are continuous, and polish. Previous to setting, hollow out the base of the crown, avoiding the edges; this provides for the presence of a body of cement between the root and the crown, as in the Logan and other crowns. Clean out the tube thoroughly and roughen its interior as in plate-work, and fix it with cement, pressing it firmly to place with a Bonwill crown-setter. The head of the pin may be riveted with an engine-burnisher, but do not omit to examine the bite before the patient leaves.

The shaping of the root is a matter of choice. The two which I have found best are the "saddle" and the well-known "New Richmond" shapes. The crown to suit the latter is best fine-fitted by hand, with a three-sided corundum file.

*Tube Crowns on Metallic Caps.*—If for any reason it is considered advantageous to protect the surface of the root by means of a metallic cap and band, the rubber dam must be dispensed with. Trim the root, making the sides parallel, and, after fitting a collar to it, leaving the gold a trifle high, prepare the canal and insert a post loosely. Next take a plaster impression and bite of the whole; the pin and band will either come away with it or, should they not do so, they can be easily replaced. Cast and open; fit a coin gold cap, No. 30 thickness, and, having soldered it to the band, through it drill or punch a hole for the pin; next place it on the model, insert the pin, and, when needed, correct its direction by bending before soldering to the cap (the pin will be easily bent if nicked with a file, and as this weakness is repaired by the soldering, it in no way imperils the soundness of the post).

Having boiled in pickle, replace the united pin, cap, and band on the model, and proceed to fit the crown. This done, cement it to cap and pin before inserting them in the mouth. This makes a strong and beautiful crown, and while it is applicable *a priori* to single-rooted teèth, it may be employed on some molars.

*Tube Crowns on Living Teeth.*—It is seldom that tube teeth can be used for this purpose, but two cases have lately been treated by me with great success. The first of these was a lower left first bicuspid, which had a large amalgam filling, extending to the crown, on each of its approximal surfaces. The tooth was much discolored, and by its presence the looks of a good set of teeth was spoiled. The patient objected to having the nerve drilled into and killed, the more when, on removing the discolored crown, calcification of the pulp was discovered. It was decided to grind down the buccal aspect of the root nearly to the gum-margin, leaving the lingual side considerably higher. A cap and band were made to fit the root tightly and pass a short distance under the gum; a pin was soldered to this, a tube tooth adjusted to it and the bite, and the whole cemented on the living root. This device has been worn for two years, and bids fair to last twenty. The buccal side of the twenty-two-carat gold band is almost covered by the gum, and what of it is seen looks like a tiny cervical filling. The second case thus treated does not differ essentially from this one.

*English Tube Teeth in Bridge-Work.*—All the points of special worth given at the beginning of this paper are emphasized when tube teeth are used in bridge-work. One of the great obstacles to making a denture of this kind a success is the difficulty met with in hiding the gold when the patient laughs. By the use of English tube teeth this is easily accomplished.

For fixed bridges replacing the front teeth they are less suitable, because of the difficulty found in securing proper self-cleansing space.

A case where the cuspids are past filling, and the molars still stand, will serve to explain the manner of constructing a large removable plate bridge with tube teeth. The crowns of the cuspids are to be cut level with the gum and the roots prepared after the usual fashion, the canals being drilled to receive a gold or platinum tube, which should be as long as possible, and sufficiently wide to accommodate a No. 13 post of hard gold. The molars are next trimmed to receive gold crowns, and a considerable notch is cut in the crown and anterior approximal surface of each. This notch (the object of which will be presently explained) should not extend on the coronal surface more than half-way back, nor on the anterior aspect more than half-way from the crown to the gum. Tubes are then placed in the roots of the cuspids and allowed to project about three-eighths of an inch. An impression of the mouth is next taken in plaster, in which the tubes will come away, and it is cast and opened as usual. The plaster teeth and roots are now trimmed, so that the cuspid caps and molar crowns when made will pass a little way beneath the gum-margin. The pattern of cap and band for the cuspid is the "Richmond," and care should be taken that each is made level with the gum on the labial side. Hard gold or platinum tubes are next soldered to them in lieu of the ordinary posts of single "Richmond" crowns. The fixing of these with cement in their proper positions, and the operation of sealing the apical ends with gold or amalgam, complete the preparation of the roots. They are then ready to receive their posts. A Melotte die of each molar is next taken, and a gold collar made to fit it. This collar is notched on its anterior surface to suit the corresponding depression on the same surface of the natural tooth; the band is put on the Melotte cast, and a piece of No. 30 pure gold is placed over the crown and burnished to fit its upper surface and the floor of the notch referred to. When this is soldered to the collar, it gives an all-gold crown without cusps. A pure gold cap is next struck up and filled in with coin gold; it is ground level on its under surface, and is in turn notched at the same part as the gold crown already made. Having adjusted it to the latter, solder together, and you have an ordinary all-gold crown, plus the recess on the crown and anterior surface.

These crowns are cemented upon their respective teeth. Posts with bent ends are now placed in the cuspid tubes and allowed to project from them about three-eighths of an inch or more. A plaster impression of the whole is now to be taken; the pins will come away in it if the direction of the cuspid tubes has been carefully considered. (Before casting the impression, slip a small piece of metal tubing of such a size as will exactly fit the posts over each; this will prevent any alteration in their direction when they have to be withdrawn and replaced in their sockets during the making of the bridge.)

Having cast, opened, and hardened the model, proceed to make the clasps for the fixed molar crowns, as follows: First take a Melotte die of each tooth and cut a pattern, being careful to leave a portion of it high enough above the level of the tooth to permit of its being bent down and accurately fitted into the notch. The clasp is next fitted to the tooth, and the high portion is thinned down with a file and punched till it fits into the depression, as just indicated. It is now strengthened and contoured to the normal shape of the crown by the addition of pieces of hard gold soldered together with twenty-one-carat solder. This forms a strong partial cap or spur, which, bearing on the gold crown, prevents the bridge's settling too hard upon the gum. It is better to make the band and spur from one piece of metal than to solder the spur to the band when fitted, for by the former way the continuity of the metal is unbroken. The clasp must be prolonged posteriorly to grasp the distal surface of the crown, in order that any tendency on the part of the tooth to backward movement, by pressure on the spur (which will thus act as an inclined plane), may be prevented.

Now proceed with the swaging of the plate, which is made of two thicknesses of metal. Make the first one No. 24 gauge and about five-eighths of an inch wide all around. It must be struck up sharply and made to cover the cuspid caps. Next take a piece of plate, No. 26 gauge, and a trifle narrower than the first, and strike it over the latter; when fitted, solder the two together with twenty-one-carat solder, and trim to shape the thick single plate thus produced. After having seen that the plate fits, drill it through opposite each cuspid tube to receive the posts, which are introduced into the tubes and allowed to project through the drill-holes on the lingual surface. Now adjust the clasps and place a little plaster around them and the cuspid posts. When it has hardened, remove the various parts from the model, stick them in their respective places, invest, and solder with twenty-carat solder.

When bands and pins have been soldered, try the bridge in the mouth and take the bite. Select suitable tube teeth, and fit them as in ordinary plate-work.

If the cuspid tube-tooth posts be thicker than the size of pin-wire, they are to be reduced by the file to suit the porcelain teeth.

Modification of this method can be used in the construction of any removable tube-tooth bridge. The point to be most noted is the treatment of the molars, a plan which can be adapted to suit any of the posterior teeth. It most surely prevents the "settling" of the denture and the tendency to movement on the part of the natural teeth.

*Fixed Bridge-Work.*—Fixed bridge-work offers but a limited scope to English tube teeth, for they can, as a rule, be used as substitutes for the masticating teeth only, for reasons which render useless the adaptation of Logan, Bonwill, and other all-porcelain crowns to like purposes. The idea must not be formed, however, that tube teeth can never be used here. In point of fact, they can, but on account of the shape of a front tooth which necessitates a short and weak lingual surface, often to be further destroyed to accommodate the bite, it has always seemed to me inadvisable to use them except in a few exceptional cases, where the bite of the lower teeth strikes abnormally far in. Here they may safely be applied. The kind of case in which a fixed bridge with English tube teeth answers admirably is one where a gap in the dental arch extends from the wisdom-tooth to the first bicuspid. The first bicuspid is banded and capped, and a pin (which acts as post both to the root and tube crown) is soldered through it. A gold crown is fitted to the wisdom-tooth, and a strong oval-shaped twenty-two-carat gold bar is made which will connect the crown and cap, and ultimately carry the teeth. This bar ought not to rest on the alveolar ridge, but must be about one-sixteenth of an inch from it, and its angle with the alveolar border ought to be such a slope downward from the lingual to the labial side as will secure a perfect self-cleansing space. The anterior end of the bar must now be soldered not only to the bicuspid cap, but also to the base of the post itself, so that the strain may be borne by both. So far, then, the bar and cuspid cap are in one piece, the molar crown remaining unattached. Place these in their relative positions in the mouth ; do any adjusting that may be necessary between the molar crown and the posterior end of the bar; take them off with plaster, as just described in this operation in plate bridge, and solder. A bite must now be taken, and the teeth set up on the bar in the usual way, being fitted to it and allowed to overhang its buccal edge. When the teeth have been cemented to place with sulphur, the bridge had best be inserted temporarily in the mouth till it has proved satisfactory, when it may be fixed. A fixed bridge like this may be inserted on either side of either jaw, and modified to suit such exigencies as intermediate roots, etc.

It must not be concluded that the possibilities of tube-work have by any means been exhausted in this essay. They are at once seen to be limited by the fault in construction of the front teeth mentioned previously.

A point of great moment to the tube-worker is the alloys of gold for posts. In plate-work these are made by English dentists about eighteen carats fine. This comparatively poor grade of metal is good enough for plate-work, but in crown- and bridge-work something finer is required. For these the qualities most to be aimed at in alloying are toughness and non-liability to tarnish. Color as indicating purity is of no importance. From experience the author recommends the use of English coin gold alloyed with from one and a half to two pennyweights of platinum to the ounce for *all pins, posts, plates, and bars.* This alloy is so infusible as to admit of soldering with coin gold twenty-two carats fine.

The use of sulphur as an agent for fixing teeth on plates and bridges is strongly advocated. Excepting where it cannot (from its very nature) be employed, sulphur is far and away the best material. It will stand in mouths which are death to the very best cements, because none of the oral foods destroy it. Again, when repairs have to be done, where cement has been the fixing medium, the teeth can only be removed with great force by pliers, obviously a very unsafe proceeding. Indeed, if the pin and tube have been well roughened, the teeth cannot be, in many cases, got off without fracturing them. By the use of the agent advised all this trouble is prevented, for when the plate comes to be prepared you have only to heat it carefully and gradually till the sulphur melts, when the teeth may be easily lifted from their pins and refixed when the repair is effected.

It may be objected by some hypercritical individual that the appearance of the pins on the coronal surface of tube teeth seriously affects their value prosthetically. This, if objected to, may be overcome very easily. Having cut as much off the pin as you think fit, without impairing its function as a support to the tooth, take a white glass or porcelain rod of proper size, and insert a piece of it in the tube over the pin. This ought to be done before the teeth are finally fixed, so that the section of glass or porcelain will be firmly held by the sulphur. When finished, the most critical observer will hardly detect any break in the color of the crowns if the inlays have been well matched.

To dentist and to patient the tube tooth yields such results as cannot be got from any other substitute. What more can an operator wish when he has presented to him for use a tooth differing essentially from the natural one in but one respect,—that it does not live? What more does the patient demand than that having his dental apparatus restored as far as utility is concerned, he is at liberty to laugh and chat to his heart's content without betraying the fact that he owes his perfect comfort not to nature, but to art? It is no exaggeration to say that in the use of the tube tooth such a happy state of things is realized. It is my opinion, formed on long experience, that were these teeth to once gain a footing in bridge-work, they would immediately find favor with all men who devote themselves to that department of our profession. The methods available to the prosthetic dentist are neither so complete nor so many as to be above improvement, both intrinsically and numerically, and in the interest of advancement I confidently recommend to you the English tube tooth.

